# Yield of Ultrasound Guided Fine-Needle Aspiration of Intraabdominal Masses and Lymph Nodes: A Prospective Cross-Sectional Study

**DOI:** 10.4314/ejhs.v31i6.20

**Published:** 2021-11

**Authors:** Simon Shiferaw Melese, Ferehiwot Bekele Getaneh

**Affiliations:** 1 Department of Radiology, Eka Kotebe general hospital, Addis Ababa, Ethiopia Radiologist; 2 Department of Radiology, Addis Ababa University College of Health Sciences, Addis Ababa, Ethiopia

**Keywords:** Diagnostic yield, Fine-needle aspiration, Ultrasonography

## Abstract

**Background:**

Ultrasonography (USG) guided fine needle aspiration cytology (FNAC) is a safe, rapid and accurate procedure for establishing diagnosis of intraabdominal lesions. The aim of this study is to assess the efficacy, and factors affecting the yield of USG guided FNAC. In addition, we intent to analyze the various intraabdominal masses based on location and cytological features.

**Methods:**

A prospective cross-sectional study was conducted in the department of radiology, between September 2019 – September 2020. Patients' preparation and USG guided FNAC procedures were performed according to the departments protocol. Chi-square test was used to assess the significance of association between independent and dependent variables.

**Results:**

Seventy-four USG guided FNAC were performed. The liver was aspirated in 34 (45.9%) followed by omentum, nine (12.2%), abdominal lymph nodes in seven (9.5%) and ovaries in four (5.4%) of the cases. The sample was diagnostically adequate in 56 (75.7%). Malignancy was diagnosed in 52 (70.3%) followed by inflammatory 3(4.1%) and one (1.4%) case of indeterminate spindle cell neoplasm (1.4%). There were no statistically significant associations between the diagnostic yield and location of the lesion, the size of the lesion, the sampling technique, the number of needle passes and qualification of the operators.

**Conclusion:**

USG guided FNAC provides a good diagnostic yield and there is no difference in diagnostic yield between the various location of the lesions, sampling techniques, number of needle passes and qualification of the operators. A larger size study is recommended to better analyze factors affecting the diagnostic yield of this procedure.

## Introduction

Intraabdominal masses are frequently encountered pathologies in clinical practice. These include a variety of neoplastic, both benign and malignant, and non-neoplastic (inflammatory) processes. They can present as superficial or deep non-palpable masses. They provide a diagnostic dilemma for clinicians and accurate diagnostic measures are important for proper patient management.

FNAC is one of a universally accepted diagnostic tool and is routinely utilized to obtain diagnostic material from these lesions. Blind FNAC has been performed frequently for superficial and palpable lesions. However, image guided FNAC is necessary to improve accuracy of tissue diagnosis for small size, deep seated and non-palpable lesions as well as for lesions located adjacent to critical structures such as neurovascular structures (1–4). Different imaging modalities like USG, Computed Tomography (CT) scan & fluoroscopy can be used for guiding the aspiration of these lesions for tissue diagnosis. USG guided FNAC is an increasingly standard diagnostic procedure for these lesions. It has been shown to correctly guide sampling of lesions as small as 1 cm. USG has the advantage of being readily available, rapid, less expensive and safe with no exposure to ionizing radiation (5). The main advantage of USG guided FNAC is constant real-time visualization of the needle tip while performing the procedure. Many studies have reported a high rate of diagnostic yield with USG guided FNAC of intra-abdominal masses. The factors that affect the diagnostic yield include size of the lesion, depth of the lesion, amount of necrotic tissue, the size and type of needle used and also on site cytopathological adequacy evaluation (4, 6–9).

The aim of this study is to assess the diagnostic yield of USG guided FNAC for cytological diagnosis of intraabdominal masses and lymph nodes, & to determine factors affecting the diagnostic yield.

## Materials and Methods

The study was conducted at Tikur Anbessa Specialized Hospital, department of radiology, between September 2019 and September 2020. The patients were referred from clinical departments in the hospital with clinical and imaging findings of intraabdominal masses. Assessment for accessibility of the lesions was performed by ultrasound. Patients who have a high risk of bleeding, were investigated for coagulopathy and those with normal coagulation profiles were appointed for USG guided FNAC. Patients with coagulopathy were referred back to the referring unit for correction of coagulation profile.

The procedure was performed by body imaging radiologists, fellows and final year radiology residents under supervision. The procedure was explained to the patients and verbal consent was obtained before each procedure. Sonoscape ultrasound machine with low (3–5MHz) and higher (8MHz) frequency probes and color Doppler capability was used to guide aspiration. The procedure was performed with an aseptic technique. The readily accessible lesion was targeted. Two types of needles were utilized mainly depending on the depth of the lesion. A 21G hypodermic needle was used for superficial lesions and 16–20G spinal needle was used for deeper lesions up to 9cm depth. A 10-ml syringe was attached to the needles for aspiration. In most cases 2–3 passes were done. Sampling was carried out by suctioning of the material (aspiration) or without suctioning (nonaspiration) techniques. The aspirated material was spread over several slides, air dried and sent to pathology laboratory after labelling with patient's details and the procedure. The cytological diagnosis results were collected from pathology department. The cytological reports were classified as neoplastic, inflammatory & indeterminate. The collected data was processed and analyzed using IBM SPSS statistics software version 25. Mean was calculated for continuous variables after normality was checked. The median was calculated when variables didn't indicate a normal distribution. Frequencies were used to summarize categorical variables. Chi-square test was used to assess the significance of association between independent and dependent variables. The level of significance was considered as P < 0.05.

**Ethical consideration**: Ethical approval to conduct the study was obtained from ethics review committee of the department of radiology before the commencement of the study.

## Results

**Background information**: A total of 74 patients were included in the study. Thirty-five (47.3%) of the patients were male while 39(52.7%) of the patients were female. The age of the patients ranges from 14 – 85 years, with a mean age of 43 ± 16.5 years (Table 1).

**Table 1:** Socio- demographic data of patients who underwent ultrasound guided fine needle aspiration

Characteristics	Mean ± SD and Number (%)
Age	43±16.5
Sex	
Female	39 (52.7)
Male	35 (47.3)
**Total**	**74**

**Imaging Evaluation**: Computed Tomography (CT) was used in the initial work up of the patients in 35(47.3%) of the patients, while 32(43.2%) patients underwent both CT scan and ultrasound. MRI was the initial modality in two (2.7%) of the patients.

The location of the lesion was in the liver in 34(45.9%) of the cases followed by the omentum in nine (12.2%) and the intraabdominal lymph nodes in seven (9.5%) of the cases. Four (5.4%) were located in the ovaries (Table 2).

**Table 2:** Distribution of lesion locations in patients who underwent ultrasound guided fine needle aspiration

Organ Aspirated	Frequency (%)
Liver	34 (45.9%)
Omentum	9 (12.2%)
Abdominal Lymph Nodes	7 (9.5%)
Abdominopelvic mass	4 (5.4%)
Ovaries	4 (5.4%)
Spleen	3 (4.1%)
Mesentery	3 (4.1%)
Pre sacral mass	2 (2.7%)
Small bowel wall	2 (2.7%)
Gallbladder	1 (1.4%)
Retroperitoneal mass	1 (1.4%)
Renal fossa	1 (1.4%)
Peritoneum	1 (1.4%)
Anterior Abdominal wall	1 (1.4%)
Stomach	1 (1.4%)
**Total**	**74(100%)**

**Figure 1:**
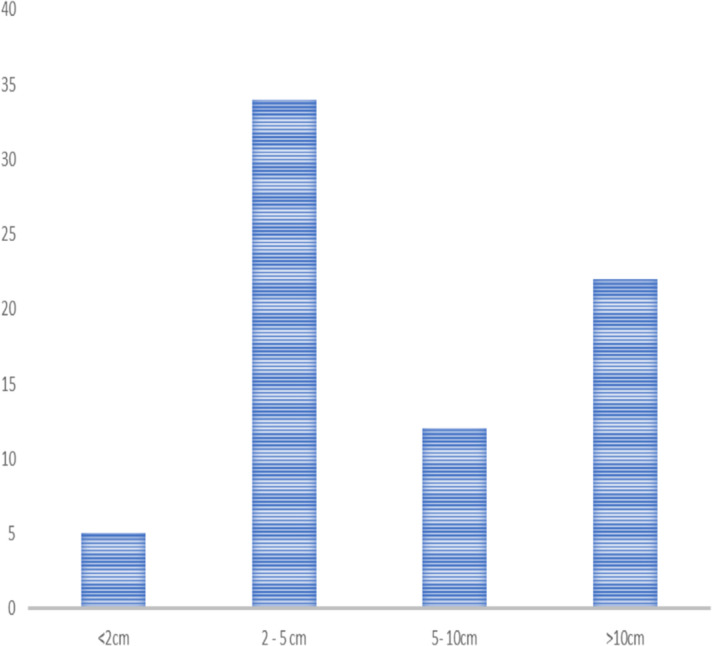
Distribution of size of intrabdominal lesions which underwent ultrasound guided aspiration

The size of the lesions sampled ranges from 2cm to 30cm with a median size of 5 (IQR=10.5) cm and 34(46.6%) the lesions had a size ranging from 2–5 cm (Figure 1).

**Sampling procedure**: From the 74 patients included, 67(90.5%) of the patients had no prior image guided procedures, while seven (9.5%) patients had a previous image guided procedure. Four of those cases underwent one previous procedure while the other three cases underwent 2 previous procedures. Two of the repeated FNAC revealed a conclusive result.

Fine needle aspiration technique was carried out for 72(97.3%) lesions. A non-aspiration and combination techniques for one (1.4%) case each. A 21-gauge hypodermic needle was utilized in 61(82.4%) of the cases, and a spinal needle was used in nine (12.2%) cases. Both types of needles were utilized in three (5.4%) of the cases. In 50(67.6%) of the cases needle passes were done three times, while in 12(16.2%) and 6(8.1%) of the cases needle passes were made two and four times respectively.

The procedures were performed by body imaging radiologist in 42(56.8%) of the cases, by radiology residents under supervision in 16(21.6%) of the cases, and body imaging fellows in 13(17.6%) of the cases (Table 3).

**Table 3:** Summary of sampling procedures performed for patients who underwent ultrasound guided FNAC

Characteristics	Variables	Frequency (Percentage)
Prior Sampling	Yes	67 (90.5%)
	No	7 (9.5%)

Sampling Technique	Fine Needle Aspiration	74 (97.3%)
	Non-Aspiration	1 (1.4%)
	Both	1 (1.4%)

Needle Type	Hypodermic Needle	61 (82.4%)
	Spinal needle	9 (12.2%)
	Both	4 (5.4%)

Number of Passes	1x	5 (6.8%)
	2x	12 (16.2%)
	3x	50 (67.6%)
	4x	6 (8.1%)
	5x	1 (1.4%)

Operator's Qualification	Radiology resident (under supervision)	18 (24.3%)
	Fellow Radiologist	13 (17.6%)
	Sub-specialist	42 (56.8%)
	Fellow under sub –specialist supervision	1 (1.4%)
	**Total**	**74 (100%)**

**FNAC yield and diagnosis:** From the 74 samples collected, 56(75.7%) of the aspirates were adequate for cytologic diagnosis while the rest, 18(24.3%) were deemed inadequate. In 10(55.6%) the reason for the diagnostically inadequate sample was a hemorrhagic aspirate. Six (33.3%) and two (11.1%) of the inadequacy was attributed to a hypo-cellular aspirates and crushed tissue sample.

FNAC diagnosis was a malignancy in 52(70.3%) of the samples followed by inflammatory in three (4.1%) cases and one (1.4%) case of an indeterminate sample reported as spindle cell neoplasm. There was no benign neoplasm reported. Results were inconclusive in 18(24.3%) of the cases.

Thirty-four (45.9%) of the fine-needle aspirations were from the liver and 28(82.35%) of them turned out adequate and malignant but six (17.65%) of the samples were inconclusive. Metastasis was reported in 12(42.85%) of them. Hepatocellular carcinoma and cholangiocarcinoma were diagnosed in 10 (37.1%) and two (7.14%) of the cases respectively. Two (7.14%) cases were small round cell tumors. Liver neuroendocrine tumor and undifferentiated carcinoma reported in one (3.57%) case each.

Aspirated lesions were located in the omentum in nine (12.2%) of the cases. Eight (88.8%) of the samples were diagnostically adequate while one (11.2%) sample was inadequate. From the adequate samples one (12.5%) of the lesion was a benign inflammatory lesion and seven (87.5%) lesions were malignant.

Seven (9.5%) of the aspirated lesions were intraabdominal lymph nodes. Five (71.4%) of them were diagnostically adequate, and two (18.6%) of the samples were inconclusive. From the adequate samples one (25%) was an inflammatory lesion while the other four (75%) were malignant. Three of the malignant lesions were diagnosed as lymphoma while the one of the lesions was a secondary adenocarcinoma.

**Factors associated with diagnostic yield**

There was no a significant difference in the diagnostic yield of the ultrasound guided procedure with regard to location of the lesion, size of the lesion, sampling technique used, number of needle passes and the qualification of the operator performing the procedures (P=0.371, P=0.078, P=0.178, P=0.574 and P=0.127 respectively).

## Discussion

Ultrasound guided FNAC is an essential procedure for acquiring diagnostic material for intra-abdominal masses. With the improvement of imaging technology, small size and deeper lesions can easily and accurately be localized with ultrasound guidance.

A broad range of diagnostic yields is reported in the literature for USG guided FNAC of intra-abdominal masses. In the present study, 56(75.7%) samples out of 74 were adequate for diagnosis. Nautiyal et al. and Nyman et al. have reported a diagnostic yield of 93.06% and 64.0% respectively (10, 11).

On cytological diagnosis, 52(70.3%) of cases were reported as malignant, three (4.1%) as inflammatory and one (1.4%) as indeterminate. A comparable finding have been reported in other studies (7, 12).

Twenty-eight (82.35%) of liver aspirates were adequate for diagnosis and all had a diagnosis of malignancy, which represent more sizeable proportion compared to other studies where they reported 77% and 68% malignancy (13, 14).

Omentum was the second most common site aspirated with nine (12.2%) cases. Eight (88.8%) of the samples were adequate for diagnosis and seven (87.5%) of which were metastatic deposits. Most studies have not reported omental lesions but Dosi et al. has reported two (1.1%) cases of omental nodules out of 174 intraabdominal masses, both of which were metastatic omental nodules (15).

Five (71.4%) of the lymph nodes aspirated were diagnostic. Four (80%) of these were malignant mainly lymphoma and 1(20%) was an inflammatory lesion. In contrary, others reported inflammatory lesions being more common, 58.8%(10/17) and 50%(4/8) respectively (12, 15).

The size of the mass, depth of the lesion, extent of tissue necrosis, the skill of the radiologist, number of passes and the size and type of the needle are some of the factors affecting yield of FNAC. Smaller lesions are reported to have a high rate of inadequacy compared to larger lesions (7). However, we found increased number of inadequate aspirates for lesions larger than 5cm. The inadequacy rate was 6/39 for lesions less than 5 cm in size as compared to 12/34 for lesions larger than 5 cm in our study.

Some studies have shown that large caliber needles provide increased cellular material but with increased risk of complications & hemorrhagic contamination (8, 9). In contrary to these findings, other study states that small caliber needles have similar or improved diagnostic yield as compared to larger caliber needles (16). In the present study, we used 16G and 18G spinal needles and 21G hypodermic needle. However, there was no significant difference in the diagnostic yield between the two groups. Although we experienced only one case where non-aspiration technique was used, some studies have shown non-aspiration technique can yield adequate diagnostic material (17–19).

We did not find statistically significant association between the diagnostic yield and the experience of the operator and the number of passes done.

The limited sample size was a limitation in this study. Therefore, larger sample size studies will help to better analyze the various factors affecting the diagnostic yield of USG guided FNAC. We also recommend on site adequacy evaluation service by pathologists for improvement of the diagnostic yield.

In conclusion, USG guided FNAC provides a good diagnostic yield. The diagnostic yield is comparable between lesions in various location, sampling techniques, number of needle passes and qualification of the operators.
